# Investigating the Extent to Which Patients Should Control Access to Patient Records for Research: A Deliberative Process Using Citizens’ Juries

**DOI:** 10.2196/jmir.7763

**Published:** 2018-03-28

**Authors:** Mary P Tully, Kyle Bozentko, Sarah Clement, Amanda Hunn, Lamiece Hassan, Ruth Norris, Malcolm Oswald, Niels Peek

**Affiliations:** ^1^ Health E-Research Centre, Division of Imaging, Informatics and Data Sciences School of Health Sciences, Faculty of Biology, Medicine and Health The University of Manchester, Manchester Academic Health Science Centre Manchester United Kingdom; ^2^ Division of Pharmacy and Optometry School of Health Sciences, Faculty of Biology, Medicine and Health The University of Manchester, Manchester Academic Health Science Centre Manchester United Kingdom; ^3^ Jefferson Center Saint Paul, MN United States; ^4^ Department of Geography and Planning School of Environmental Sciences University of Liverpool Liverpool United Kingdom; ^5^ Health Research Authority London United Kingdom; ^6^ School of Law Faculty of Humanities The University of Manchester Manchester United Kingdom; ^7^ Citizens Juries Community Interest Company Manchester United Kingdom; ^8^ Greater Manchester Patient Safety Translational Research Centre Division of Population Health, Health Services Research and Primary Care, School of Health Sciences The University of Manchester, Manchester Academic Health Science Centre Manchester United Kingdom

**Keywords:** public participation, patient engagement, public opinion, medical research, confidentiality, privacy, national health services, data linkage, public policy, decision making, organizational

## Abstract

**Background:**

The secondary use of health data for research raises complex questions of privacy and governance. Such questions are ill-suited to opinion polling where citizens must choose quickly between multiple-choice answers based on little information.

**Objective:**

The aim of this project was to extend knowledge about what control *informed* citizens would seek over the use of health records for research after participating in a deliberative process using citizens’ juries.

**Methods:**

Two 3-day citizens’ juries, of 17 citizens each, were convened to reflect UK national demographics from 355 eligible applicants. Each jury addressed the mission “To what extent should patients control access to patient records for secondary use?” Jurors heard from and questioned 5 expert witnesses (chosen either to inform the jury, or to argue for and against the secondary use of data), interspersed with structured opportunities to deliberate among themselves, including discussion and role-play. Jurors voted on a series of questions associated with the jury mission, giving their rationale. Individual views were polled using questionnaires at the beginning and at end of the process.

**Results:**

At the end of the process, 33 out of 34 jurors voted in support of the secondary use of data for research, with 24 wanting individuals to be able to opt out, 6 favoring opt in, and 3 voting that all records should be available without any consent process. When considering who should get access to data, both juries had very similar rationales. Both thought that public benefit was a key justification for access. Jury 1 was more strongly supportive of sharing patient records for public benefit, whereas jury 2 was more cautious and sought to give patients more control. Many jurors changed their opinion about who should get access to health records: 17 people became more willing to support wider information sharing of health data for public benefit, whereas 2 moved toward more patient control over patient records.

**Conclusions:**

The findings highlight that, when informed of both risks and opportunities associated with data sharing, citizens believe an individual’s right to privacy should not prevent research that can benefit the general public. The juries also concluded that patients should be notified of any such scheme and have the right to opt out if they so choose. Many jurors changed their minds about this complex policy question when they became more informed. Many, but not all, jurors became less skeptical about health data sharing, as they became better informed of its benefits and risks.

## Introduction

### Public Opinion on Data Use

The last decade has seen a surge in the reuse of data that were created for the health care of individual patients for additional purposes such as for research (so-called secondary use of data). Research studies that previously would have been impossible because of the effort required to collect data have now become possible by reusing data originally collected for the purposes of providing direct health care. Examples include investigations into the prescribing of anxiolytics and hypnotics in over 300,000 children and young people in Wales [1], the mental health of 57,000 veterans compared with173,000 nonveterans in Scotland [2], and the impact of a smoke-free legislation on stroke [3].

In many countries, there is no lawful impediment to the use of deidentified (or anonymized) data for research without the consent of the data subject, as long as the risks of reidentification are very low or remote. This may include the linkage of data from multiple sources before deidentification. However, public support for such research use of data without consent, the so-called social license, is separate from any legal framework [4]. Even where no legislation exists to prevent the use of deidentified data, the lack of a social license may ultimately result in the failure of data-use initiatives, as has been the case with national data records systems in England [4] and Australia [5].

Epidemiologists are dependent upon using data without consent for such research for numerous reasons. Obtaining consent from many thousands of people is an onerous task, and there is a strong likelihood of many people being disinterested and not giving consent simply because they do not remember being asked [6]. Large amounts of missing data, which are often from particular subgroups rather than randomly distributed through the population, can mean that findings from epidemiological studies can be misleading [7].

Despite such opinions in the aggregate, individual public attitudes toward the secondary use of data vary [8,9]. In particular, public support may be different, depending on who is using the data or the use to which the data may be put. Such reuse of data without consent is an area of concern to some members of the public [10]. These people express the wish to be asked to consent to every use of the data, whereas others want to give a general consent for data use. Still others are content with the data being used without them being aware, consulted, or asked at all, provided that the research has been reviewed and approved by an ethics committee [9].

### Deliberative Approaches

It could be argued that, for such a complex area, surveys may not be the best method to find out about the decisions that the public would make. Members of the public are often unaware of the ways that data are used and the governance procedures that are put in place to protect health care data [8,10,11]. Thus, it could be surmised that some survey respondents do so from a position of ignorance of the topic. Qualitative methods such as focus groups or deliberative processes provide a more nuanced view of public opinion. Some of these methods, particularly those which employ deliberative approaches, enable questions to be answered about what citizens would think regarding the use of data if they were informed. Few studies have done this [11,12]. Recently, the Wellcome Trust compared findings from a survey about the commercial use of data conducted with 2017 members of the public, with findings from 16 focus groups with 246 people [11]. The focus groups indicated how people change their minds once they were slightly more informed about the use of data through discussion with their peers in the group, whereas surveys are generally conducted at a single point in time.

This suggests that there is much to be learned from using deliberative methods that allow participants to learn about and reflect on information about such a complex area. Citizens’ juries are comprehensive engagement processes that allow decision makers and the public to hear thoughtful input from an informed microcosm of the public [13]. They are based on the premise that, given enough time, opportunity, support, and resources, members of the public are quite capable of arriving at decisions about complex matters [13,14]. The citizens’ jury process is designed to allow decision makers to hear citizens’ voices. It provides an opportunity for citizens to learn about an issue and deliberate together to find a common ground solution. Decision makers can thus learn more about what an informed public wants and why they want it [13].

There are examples of organizations using citizens’ juries to help make policy decisions, even though members of juries are not elected and cannot be made accountable for decisions. For example, Melbourne City Council has appointed a citizens’ jury to determine how to allocate its 10-year Aus $5 billion budget, and the council is implementing virtually all of the jury’s recommendations [15].

### Study Aim

The aim of this study was to investigate what people think about secondary use of data, including data linkage, once they become more informed about the area. The outcome of the citizens’ juries was to inform the ongoing research, information governance, and public engagement strategies of the project’s sponsors: the UK's national Farr Institute of Health Informatics Research [16] and the Greater Manchester Primary Care Patient Safety Translational Research Centre [17].

## Methods

### Jury Process

The citizens’ juries were run over 3 days as jury pairs [18], that is, two juries were conducted in the same geographical area, addressed the same jury mission (Textbox 1), listened to the same witnesses, but were comprised of different people. To ensure that the juries were conducted appropriately, the manual written by the Jefferson Center, the developers of the method, was followed [13], and the juries were run by an experienced facilitator from that center (KB). Approval for video recording of the jury discussions was obtained from The University of Manchester’s research ethics committee.

### Jury Recruitment

The citizens’ jury process uses members who are selected to be representative of the population in key criteria [13]. Recruitment questionnaires collected data to enable selection against *a priori* criteria based on demographics and views on privacy (Table 1). The demographics provided a broadly representative sample of resident adults in England based on the 2011 census with respect to gender, age range, ethnicity, and educational attainment [19]. Potential participants were asked to complete an Ipsos MORI survey question [20] that involved balancing privacy against information sharing for public benefit (Textbox 2). This was the most up-to-date survey of public opinion in this area at the time of recruitment. The demographic and privacy criteria insured that each jury was a “microcosm of the public” [13].

**The jury mission.**

Suppose a National Health Service (NHS) body wants to create new records from the patient records stored by your general practice and by hospitals that have treated you. They want to use them for purposes other than your direct patient care, such as research about better treatments and for checking that patients are receiving safe and effective health care. These records would be held securely and would not contain your name, address, and other identifiers. Despite this, there is a small risk that the records might still identify you because they would contain lots of detailed information about the care you receive from your general practitioner and from different hospitals. The NHS body would also review requests from other public and private organizations, granting access only where they believed it was lawful and in a good cause.

1.
  - Should the NHS body be allowed to create these records about you and other patients? (choose only one of the following)
    - Yes, but they should publish information about what they plan to do
    - Yes, but they should publish information about what they plan to do and patients should be able to opt out
    - Yes, but they should publish information about what they plan to do and only create records for patients who opt in
    - No
    - Other (explain in less than 30 words)
  - Give reasons for your answer (in less than 300 words)
2.
  - Given your answer to question 1, who should be allowed to access and extract data from the records created? (Choose as many of the following examples that apply)
    - NHS clinicians and administrators who decide which health services should (and should not) be funded
    - NHS clinicians and administrators doing approved research into whether doctors are prescribing medicines appropriately
    - University staff doing approved research into whether doctors are prescribing medicines appropriately
    - Staff employed by local authorities planning the future need for residential care homes
    - Staff employed by a private company being paid by a hospital NHS trust to compare the number of people dying after surgery with other hospitals
    - Staff employed by an insurance company aiming to set health insurance premiums accurately
    - Staff employed by a pharmaceutical company investigating whether they should begin research into a new drug for a genetic disease for which there is currently no treatment
  - Give reasons for your answer (in less than 400 words)

**Table 1:** A priori criteria for jury selection and demographics of actual jurors.

Criteria		UK census (%)^a^	Jury target range	Achieved in jury 1 and 2^b^
**Gender**				
	Women	51	8-10 jurors	8 and 9 jurors
	Men	49	8-10 jurors	9 and 8 jurors
**Age range (years)**				
	18-29	21	2-5 jurors	5 and 3 jurors
	30-44	26	3-6 jurors	4 and 6 jurors
	45-59	25	3-6 jurors	5 and 5 jurors
	60+	28	4-7 jurors	3 and 3 jurors
**Ethnicity**				
	White	85	14-17 jurors	14 and 14 jurors
	Groups other than white	15	2-4 jurors	3 and 3 jurors
**Educational attainment**				
	Level 1 or no qualifications	36	5-8 jurors	6 and 7 jurors
	Level 2 or level 3 qualifications (apprenticeship and other qualifications)	37	5-8 jurors	6 and 5 jurors
	Level 4 qualifications (degree level) and above	27	4-6 jurors	5 and 5 jurors
**Privacy views^c^**				
	Agree more with a) than b)	52	7-11 jurors	9 and 10 jurors
	Agree more with b) than with a)	34	5-7 jurors	5 and 6 jurors
	Agree equally with both or don’t agree with either or don’t know	14	1-4 jurors	3 and 1 jurors

^a^[19].

^b^1 person left each jury at the end of the first day and are not reported here.

^c^Target sample percentages based on “Perceptions of Data Sharing” survey [20]—see Textbox 2 for full text.

**Ipsos MORI survey question used to assess views and privacy for jury selection and after the jury was completed.**

As you may know, different government departments and services collect data about individuals, for example, your tax records and health records. People have different views on how much of this information should be shared within government. Data sharing can bring benefits such as finding more effective medical treatments, using information about local communities to plan local schools or roads, etc. But some people worry that data sharing will be a risk to their privacy and security, by linking different types of data together and potentially allowing them to be identified. Overall, which of the following statements is closest to your view?

- We should share all the data we can because it benefits the services and me—as long as I can opt out if I choose
- We should not share data as the risks to people’s privacy and security outweigh the benefits

1. Agree much more with a) than with b)
2. Agree a little more with a) than with b)
3. Agree equally or don’t agree or don’t know
4. Agree a little more with b) than with a)
5. Agree much more with b) than with a)

Jury members were recruited using a variety of methods to ensure that the criteria were met. Adverts were placed on websites for employment opportunities and research volunteers, emails were sent to a range of community groups, and in-person presentations were made to groups of retired people. Most of the members were recruited from the employment website. From a jury pool of 355 eligible applicants, 18 jurors and 4 reserves were selected for each jury, as recommended by the Jefferson Center [13]. Candidates meeting the criteria in Table 1 were shortlisted and interviewed by telephone to check eligibility, namely, older than 18 years; fluency in English; the capacity to contribute to jury discussions; not a health care professional; at least a year as a resident of Greater Manchester; and no special knowledge, interest, or conflict of interest in the jury mission. Jurors were thus chosen to ensure that they had “no special axe to grind” [21].

Reserves attended the jury meeting and stayed until lunchtime on day 1. In each jury, one was needed to replace a juror who did not attend or who had left during the first morning. Both jurors and reserves were paid for their time. One person withdrew from each jury at the end of day 1 for personal reasons. As the reserves had not attended for the afternoon of day 1, and therefore, had not heard the information presented by the witnesses, these jurors were not replaced, leaving 17 people to complete each jury.

### Jury Process

The jury mission was planned, designed, and refined over a period of 9 months by a project board comprising five of the authors. The jury mission asked jurors to suppose that a National Health Service (NHS) body wanted to create new records by linking data from the patient records stored by their general practice and by hospitals that have treated them. The new records were for purposes other than direct patient care, including research and service improvement. The jurors were then asked whether this should be allowed and, if so, who should be allowed access to the data. The mission was developed iteratively by the project board to reflect the question on the extent to which patients should control access to patient records (Textbox 1). Both 3-day juries followed the same program (Textbox 3). The activities were designed primarily by the Jefferson Center in line with their citizens’ jury method [13] and were managed by two facilitators who were independent of the project board and jury sponsors (KB and AH).

Five expert witnesses were chosen to provide relevant information to and answer any questions from the members of the jury (Table 2). Two witnesses were selected to provide impartial information on day 1, including about the use of deidentified data using the Information Commissioner’s Office’s (ICO’s) anonymization code of practice [22]. The code emphasizes that understanding anonymization means understanding what personal data is, that it can be impossible to assess reidentification risk with absolute certainty, and that different forms of access to anonymized data can pose different reidentification risks (eg, publication is more risky than limited access). Three advocates, known as partial witnesses, were chosen to provide arguments for and against the greater use of patient records on day 2. The purpose of the expert witness presentations was “to inform and educate the jurors, a microcosm of the public, to enable them to reach wise and thoughtful conclusions” [13], rather than to produce. Impartial expert witnesses were asked to confine their presentations and answers to questions to matters of fact rather than values. Partial expert witnesses were asked to make the case for a particular viewpoint or viewpoints based on both facts and values, and an ethicist was asked to provide arguments pulling in both directions (Table 2; see Multimedia Appendix 1 for presentations). The difference between the two types of witnesses was explained to the jurors. After each presentation, there was an opportunity for questions.

**The program of activities for both citizens’ juries.**

Day 1:

- Participants complete the start-of-Jury questionnaire and consent form
- Introduction to the event
- Group work simulation exercise (about allocation of ambulance services)
- Presentation and questions with expert witness on patient records (Ralph Sullivan), and group work to identify key learning points
- Presentation and questions with expert witness on the law (Dawn Monaghan), and group work to identify key learning points

Day 2:

- Presentation and questions with expert witness arguing for greater use of patient records in the public interest (John Ainsworth), and group work to identify key learning points
- Presentation and questions with expert witness arguing for protection and patient control of patient records (Sam Smith), and group work to identify key learning points
- Presentation and questions with expert witness identifying ethical considerations (Søren Holm), and group work to identify key learning points
- Group work to identify, discuss, and rank reasons for and against the different components of question 1 of the jury mission
- Juror voting on question 1

Day 3:

- Group work with prepared information to develop the case for and against different parties gaining access to records, as set out in question 2 of the jury mission
- Group work to identify, discus, and rank reasons for and against the different parties identified in question 2 of the jury mission
- Juror voting on question 2
- Participants complete the end-of-jury questionnaire

**Table 2:** Perspectives taken and information provided by impartial and partial witnesses who presented to both juries.

Witnesses		Perspective taken	Information provided
**Impartial witnesses**		
	Dr Ralph Sullivan, general practitioner and medical informatician	To explain what is in a patient record, and how patient records are used in the NHS.	General Medical Council requirements for record keeping, content of multiple patient records, and how they are used in practice both for direct care and secondary uses
	Dawn Monaghan, group manager for public services at the Information Commissioner’s Office	To tell jurors a little about the law that protects access to patient records.	Outline of relevant privacy law, (common law duty of confidence and Data Protection Act 1998), how data are protected, and limitations to access to data
**Partial witnesses**		
	Dr John Ainsworth, senior research fellow at the University of Manchester	To argue that it’s important that patient records are used for research and other purposes that bring benefits to the public.	How data are used to create medical evidence as to the effectiveness and safety of treatment in the public interest
	Sam Smith, medConfidential coordinator	Oo make the case for stronger control over access to patient records and better information and choices for patients about the use of patient records.	Risks of reidentification, differences between opt out and opt in, uses of data for decommissioning services, and misuse by commercial companies. Argued the case for greater control of patient records
	Professor Søren Holm, professor of bioethics at the University of Manchester	Ethical arguments for patients controlling access to patient records, and ethical arguments for wider use of patient records for the benefit of the public.	Potential benefits of sharing data, problems with sharing data, and difficulties with specific informed consent models. How these conflicting interests can be reconciled. Identified ethical considerations both for patients sharing and for patients controlling patient records for uses other than direct patient care

Jury deliberations occurred in small groups after each presentation and before the preparation of each section of the final report. The small groups recorded and reported the results of their deliberations back to the entire jury. During this time, there were opportunities to seek clarification on points of fact from the experts. In addition, if points had been misunderstood by individual jury members, other jurors corrected them. Over half of the total jury time was devoted to jury deliberations in small groups or together as a large group.

To monitor and minimize bias, an independent oversight panel was appointed. The panel members were chosen from national organizations for their subject knowledge and lack of conflict of interest: the chair of the Confidentiality Advisory Group [23], the assistant director of the Nuffield Council on Bioethics [24], and a senior policy officer from the ICO [25] with responsibility for health data. The panel reviewed the citizens’ jury design, the choice of expert witnesses, and much of the detailed jury documentation, including the jury questionnaires and the slides from the presentations by the impartial expert witnesses, resulting in some changes to these materials.

Additional design controls used to monitor and minimize bias included that the project board was only able to influence the jury mission and was independent from the jury process and outcomes. A day-long pilot workshop was conducted with seven members of the public to test aspects of the jury design, including presentations by two of the expert witnesses, some of the planned jury activities, and the pre-and postquestionnaires. This highlighted a number of issues, leading to design changes. During the two juries, jury members were asked to complete a questionnaire at the end of each day as to whether the jury facilitators or anyone else had tried to influence them toward particular conclusions. Paired juries were conducted to reduce bias and validate outcome [18]. Finally, the detailed jury design and results documentation were published online [26].

### Jury Questionnaires and Reports

Jurors were asked to complete a questionnaire at the start of the jury to identify their prior views and again after all the jury deliberations were complete (Tables 3 and 4). Data were entered into Excel (Microsoft) and collated using simple counts [13].

During the second half of the jury proceedings, the lead facilitator constructed the juries’ report with each jury. The two juries voted on individual aspects of both of the jury mission questions (Textbox 1 and Table 3). Jurors also suggested reasons for and against the jury mission options, and the most important reasons given were chosen by juror voting. Each juror had three votes that could be allocated to two or three of the reasons (no reason could get all three votes). This voting method is now the standard approach of the Jefferson Center, although not described in the manual published in 2004 [13]. It allows jurors to choose more than a single option, which is often desired when faced with a large number of possible selections. These votes and ranked reasons formed the basis of the jury reports. On the afternoons of day 2 and day 3, the facilitator led the jurors through the jury report displayed on a screen, editing in real time in discussion with the jurors to gain their acceptance that it fairly represented their views.

All jurors and reserves consented in writing for the main group deliberations to be video recorded; small group deliberations were not recorded. Eleven jurors from jury 1 also consented to be interviewed briefly on video about their views on the jury mission, to be used in a video about the jury findings [26]. Each interview lasted approximately 3 min. Sections of the videos pertaining to decision making, and the jury report were watched repeatedly by the lead author (MPT). These discussions were compared with the final versions of the two jury reports that had been prepared contemporaneously with the discussions. Relevant portions of the videoed discussions were transcribed, and verbatim quotes were selected for inclusion to highlight the discussion content. Additional explanations are provided in the quotes inside square brackets, where needed for clarity.

## Results

### Jury Process

The majority of jurors reported in their postjury questionnaires that there was no evidence of bias in the conduct of the juries. However, bias was reported by a few jurors, particularly regarding what they perceived as the impartiality of information from expert witnesses. Differences in quality of presentations by the witnesses were interpreted by one juror as a deliberate attempt to manipulate proceedings. In addition, one jury member explained in their questionnaire:

The roles of the expert witnesses made them naturally inclined to imply certain things, although nothing was explicitly said to persuade us.

The majority of jurors reported participation in the jury process to be very interesting (12 in jury 1 and 17 in jury 2) or mostly interesting (4 members of jury 1). Throughout, the jury members were fully engaged in the process of deliberation, as was evident from the videos of the proceedings, and the quality of the report that the jurors and facilitators produced.

### Jury Questionnaires

**Figure 1:**
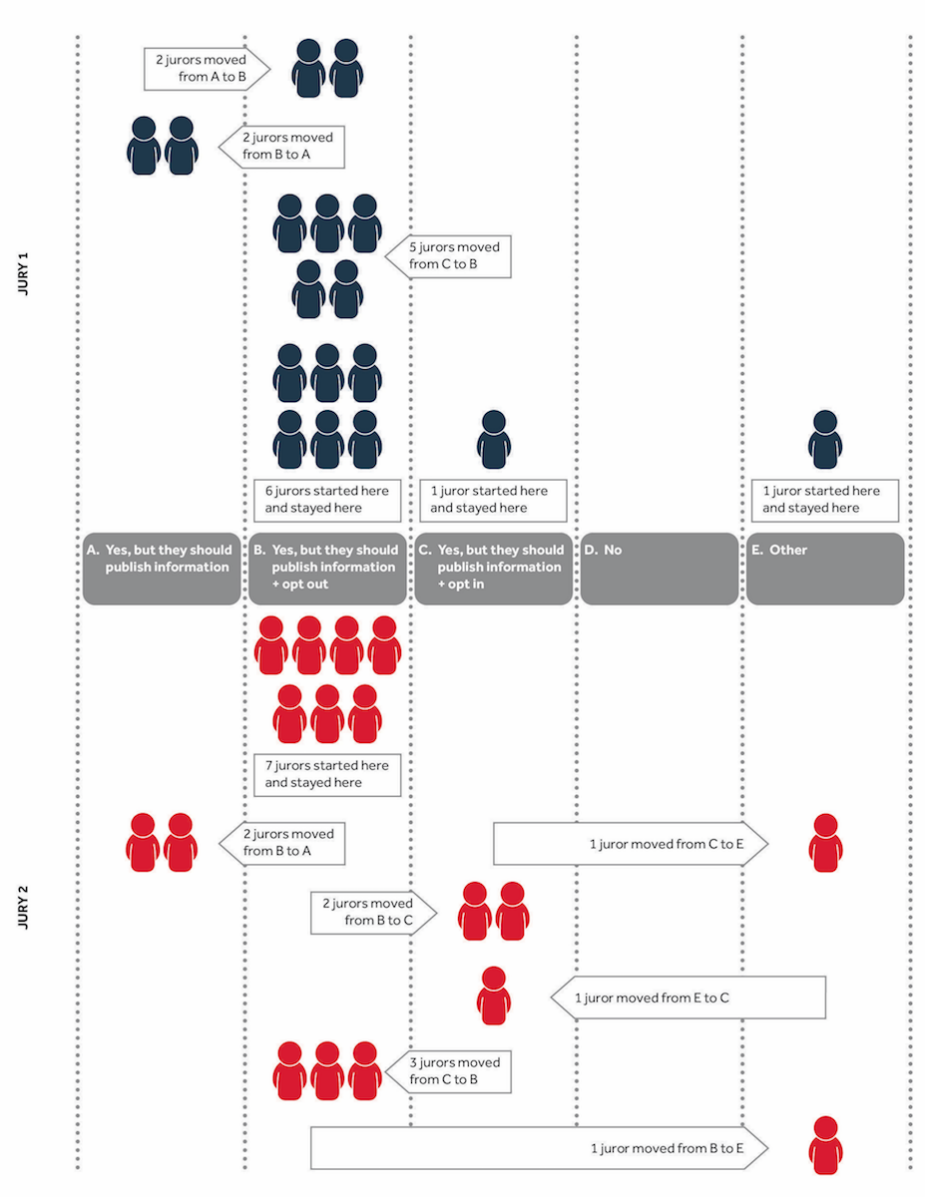
Numbers of jurors who changed their answers to question 1 of the jury mission.

Jurors completed the pre- and postjury questionnaires individually (Tables 3 and 4). In jury 1, although 8 jurors did not change their views, 9 jurors did, with 5 of them making shifts in a way that favored public benefits over privacy. In jury 2, although 7 jurors did not change their views at all, 10 did, although the shifts were not as marked as for jury 1. Although 6 jurors moved toward favoring public benefit, 2 moved more toward favoring increased privacy. Figure 1 highlights the changes in opinions of jurors.

At the end of the juries, 33 out of 34 jurors voted independently in support of the secondary use of data, with 24 wanting individuals to be able to opt out and 6 favoring opt-in arrangements (Table 3). The remaining 3 wanted data users only to publish their intentions, with no opportunity for either opting in or out. The *other* suggestions that were given by the jurors were additional requirements as to what data users should be required to do. These included giving opt-out options to children at 16 years and requiring an additional strong regulatory body. One juror wrote on their questionnaire, “I feel if it was an opt-out, people or organisations would just brush over it. Whereas if they want the numbers, it will have to be thought about and [the] public educated.” The reasons for opinion changes were not explicitly ascertained from the jurors, but some volunteered information. One juror expressed new concerns and suspicions as to the rationale for conducting data linkage and the role of the citizens’ jury in giving legitimacy to that process.

Many jurors changed their opinion about who should get access to these records, with more people supporting information sharing to a wider group of people by the end of day 3 (Table 4). The aggregate numbers in the Table belie the fact that individual jurors changed their minds in opposite directions. Four jurors in jury 1 changed their minds about NHS researchers accessing data, with 2 agreeing prejury but not postjury, and 2 agreed to allow access postjury but not prejury. Similarly in jury 2, 6 jurors changed their view regarding both university researchers and local authorities, with 3 moving from denying to allowing access and the other 3 moving in the opposite direction.

**Table 3:** Results from pre- and postjury questionnaires for jury mission question 1 completed individually by jurors, including changes in opinions. “Change” indicates previous answer to new answer. NHS: National Health Service.

Question and answer options		Jury 1 (n)				Jury 2 (n)			
		Prejury	Change	Postjury	Change	Prejury	Change	Postjury	Change
**Should the NHS body be allowed to create these records about you and other patients?**									
	a. Yes, but they should publish information about what they plan to do	2	a→b (2)	2	b→a (2)	0		2	b→a (2)
	b. Yes, but they should publish information about what they plan to do and patients should be able to opt out	8	b→a (2); b→b (6)	13	a→b (2); b→b (6); c→b (5)	12	b→a (2); b→b (7); b→c (2); b→e (1)	10	b→b (7); c→b (3)
	c. Yes, but they should publish information about what they plan to do and only create records for patients who opt in	6	c→b (5); c→c (1)	1	c→c (1)	4	c→b (3); c→e (1)	3	b→c (2); e→c (1)
	d. No	0		0		0		0	
	e. Other	1	e→e (1)	1	e→e (1)	1	e→c (1)	2	b→e (1); c→e (1)

**Table 4:** Results from pre- and post-jury questionnaires for jury mission question 2 completed individually by jurors, including changes in opinions. “Change” indicates previous answer to new answer. Y=organization should be granted access; N=organization should not be granted access. NHS: National Health Service.

Question and answer options				Jury 1 (n)					Jury 2 (n)			
			Prejury		Change	Postjury	Change	Prejury		Change	Postjury	Change
**Which organizations should be granted access to these records? (Choose all that apply)**											
	**NHS clinicians and administrators who decide which health services should (and should not) be funded**											
		Yes	10		Y→Y (10)	15	Y→Y (10); N→Y (5)	7		Y→Y (7)	17	Y→Y (7); N→Y (10)
		No	7		N→Y (5); N→N (2)	2	N→N (2)	10		N→Y (10)	0	
	**NHS clinicians and administrators doing approved research into whether doctors are prescribing medicines appropriately**											
		Yes	15		Y→Y (13); Y→N (2)	15	Y→Y (13); N→Y (2)	14		Y→Y (14)	17	Y→Y (14); N→Y (3)
		No	2		N→Y (2)	2	Y→N (2)	3		N→Y (3)	0	
	**University staff doing approved research into whether doctors are prescribing medicines appropriately**											
		Yes	9		Y→Y (8); Y→N (1)	15	Y→Y (8); N→Y (7)	14		Y→Y (11); Y→N (3)	14	Y→Y (11); N→Y (3)
		No	8		N→Y (7); N→N (1)	2	Y→N (1); N→N (1)	3		N→Y (3)	3	Y→N (3)
	**Staff employed by local authorities planning the future need for residential care homes**											
		Yes	4		Y→Y (3); Y→N (1)	10	Y→Y (3); N→Y (7)	6		Y→Y (3); Y→N (3)	6	Y→Y (3); N→Y (3)
		No	13		N→Y (7); N→N (6)	7	N→N (6); Y→N (1)	11		N→Y (3); N→N (8)	11	N→N (8); Y→N (3)
	**Staff employed by a private company being paid by a hospital NHS trust to compare the number of people dying after surgery with other hospitals**											
		Yes	5		Y→Y (5)	10	Y→Y (5); N→Y (5)	1		Y→Y (1)	6	Y→Y (1); N→Y (5)
		No	12		N→Y (5); N→N (7)	7	N→N (7)	16		N→Y (5); N→N (11)	11	N→N (11)
	**Staff employed by an insurance company aiming to set health insurance premiums accurately**											
		Yes	2		Y→Y (1); Y→N (1)	3	Y→Y (1); N→Y (2)	0			1	N→Y (1)
		No	15		N→Y (2); N→N (13)	14	N→N (13); Y→N (1)	17		N→Y (1); N→N (16)	16	N→N (16)
	**Staff employed by a pharmaceutical company investigating whether they should begin research into a new drug for a genetic disease for which there is currently no treatment**											
		Yes	7		Y→Y (7)	12	Y→Y (7); N→Y (5)	5		Y→Y (4); Y→N (1)	10	Y→Y (4); N→Y (6)
		No	10		N→Y (5); N→N (5)	5	N→N (5)	12		N→Y (6); N→N (6)	7	N→N (6); Y→N (1)

**Table 5:** Votes on subquestions of the jury mission completed during the writing of the jury report. NHS: National Health Service.

Questions and answer options		Jury 1	Jury 2
**Vote 1A. Should the NHS body be allowed to create these records about you and other patients?**			
	Yes	17	13
	No	0	4
**Vote 1B. If such records were created, should they only publish information about what they plan to do or allow a patient option (type unspecified)**			
	Publish only	8	5
	Patient option	9	12
**Vote 1C. Should individuals have the option to opt in or opt out?**			
	Opt in	1	5
	Opt out	16	12

### Jury Reports

In addition to the questionnaires above, jurors voted separately on three individual aspects of question 1, by private poll, during the writing of the report with the facilitator (Table 5). Voting on each subquestion was undertaken over the afternoon of day 2 (vote 1A) and throughout day 3 (votes 1B and 1C). As a consequence, jurors could and did change their opinions during later votes without being able to change the vote they had given for earlier questions. Thus, in jury 1, for example, only 9 jurors voted for patient input in vote 1B, but all were required to choose between two different types of patient input in vote 1C. The reasons for changing their minds could be either based on individual rights or pragmatism. Two jury members from jury 1 stated:

I changed my mind following this discussion, yeah, well I would have said that they should just announce it [publish] because I thought if they are going to do it [use data], do it so it is accurate and everybody is included in it. But now I am thinking, well human rights, should people have a decision, you know, whether to be included or not, yeah.

I was thinking publish but, in reality, if we give people an option, I don’t think they will opt out as much, but if you don’t give them an option most people will go off [get angry].

### Use of Data

In vote 1A, all jurors in jury 1, and all but 4 jurors in jury 2, voted that the NHS should be allowed to create linked records (Table 5). The reason most commonly voted for by both juries was that more detailed and complete data would produce more accurate evidence, which can lead to more effective, more cost-effective health care through the NHS (see Multimedia Appendix 2). Other reasons included that personalized medicine and treatments could only be discovered and used effectively through use of more complete data and records and that data use would help identify ineffective drugs and treatments sooner so that they can be removed from use and increase patient safety.

The main argument mentioned by most jury members against creating and sharing linked patient records (regardless of whether they voted in favor of still doing it) concerned transparency of use. They felt that, without a clear understanding of who would be regulating the data and making decisions about access, it was difficult to support the creation of new records. In addition, there were concerns that this would benefit researchers or companies rather than individual patients, as explained by this juror from jury 2:

What proof is there that the general public would be any better off in terms of that research with all the data being in one place, then? Whereas at the moment there is all this research going on anyway, but it is more difficult for the researchers and the private companies to get the information, because they have to go all over for it. So is there any evidence to prove that it would be better than what it is at the moment?

There were concerns that despite safeguards, data and records may not be secure and may be accessed by individuals or organizations without proper permission or legal authority, or for reasons other than where originally authorized. In addition, there were concerns that data may be used by private companies for commercial gain rather than for the benefit of patients and the public, or sold on to other companies.

### Patient Choice

The issue of whether there should be patient choice (either opt out or opt in), or whether the NHS body should merely follow the minimum legal requirements of publishing that data use had taken place, divided both juries. In jury 1, 8 people voted to publish only, whereas in jury 2, only 5 people voted for this. The reasons given were that this would ensure more accurate, complete data when all records are included, which would be of greater benefit to the population and that it would save time and money through a much more streamlined, efficient process. Reasons given why patients should have input included that there was an expectation that people should be able to have autonomy and freedom of choice by having control over their own data and records and choosing whether or not their record is included and that it would allow individuals to maintain their confidence in doctors and other health care settings where trust is critical. In addition, it was suggested that the process of obtaining patient input would allow greater transparency in how records are used and shared.

Jury 1 had more members suggesting an opt-out model in comparison with jury 2. The most frequently selected reason they gave for suggesting this model was that more people would be included in the data, and this would lead to more accurate results and more representative samples of the population, and this in the end would lead to more rigorous research and better treatments. Other reasons included that this would be more effective in terms of time and money, as it was an easier and more convenient option for individuals. The alternative would take an enormous effort and may still not properly provide the opportunity to every individual to make an informed decision. This option would allow those who may simply be undecided (but not opposed) to still contribute to research and improvements in health care. The reasons that jurors suggested for an opt-in option included that this option would require the organization to conduct an information campaign to educate the public and would mean that individuals whose data were used in analysis could make an informed decision to be included.

### Data Access

When considering who should get access to data, the two juries had very similar rationales, which were written in the report that they produced at the end of the third day. Both thought that public benefit was a key justification for access. Jury 1 was more strongly supportive of sharing patient records for public benefit, whereas jury 2 was more cautious and sought to give patients more control. In particular, they concluded that organizations and individuals who *should* be granted access to these records tend to demonstrate similar characteristics. Typically, these organizations clearly demonstrated that the primary goal for using the data was for public benefit (such as improved medical care and treatments, improved public health, or management of public funds) and made a clear and compelling case for why they need these patient records. They provided clear justification for how and why the data would be used, why it was relevant to their efforts, with whom it will be shared, and only access records they needed to perform their data analysis and could not get adequate data from other sources. The organizations showed a clear, relevant connection between the issues they are addressing and the information contained in these records, had a track record of protecting data and records, and could be trusted to maintain control of data without sharing and have controls in place to properly secure the data and safeguard against internal misuse. Finally, these organizations needed access to the data to conduct urgent and/or timely analysis.

The reasons the juries gave for why organizations should not have access to the data included several that were the opposite of the reasons for access, such as organizations that did not clearly indicate that the primary use of the data is for public benefit, who may use the data solely for private gain or commercial profit, or who did not have a trusted track record for protecting data. In addition, they might use the data to exploit or manipulate individuals or populations or might manipulate the data to support their own agenda.

## Discussion

### Principal Findings

The findings of the citizens’ jury work highlight that, when informed of both the risks and opportunities associated with health data sharing, members of the public believe an individual’s right to privacy should not prevent research that can benefit patients overall. The juries also concluded that patients should be notified of any such scheme and have the right to opt out if they so choose. Many, but not all, jurors became less skeptical about health data sharing, as they became better informed of its benefits and risks.

### Attitudes to Data Use

The findings from this study support the contention that some members of the public believe that NHS records are a public resource, paid for by public money, and therefore, should be used for research for the public benefit [27,28]. Few jurors objected to the use of health data *per se*, but many wanted, as a minimum, to be told that such uses were happening and to be given an option to opt out. It reinforces the fact that the social license, or the societal expectations as to how deidentified data should be used, is not necessarily the same as what is permissible by law [4].

There were both individual and aggregate changes in attitude, which has been found in some [8] but not all [29,30] previous studies in this area. The jury members had the opportunity to learn about and deliberate on the general use of linked health data over the course of 3 days, which may well have contributed to how they changed their opinions. For most of the jury members, this change related to becoming more accepting of less patient control over the use of data or more pragmatic about the need for slightly more patient control, depending on their initial views. However, two jury members changed their mind quite strikingly and became much more insistent upon greater patient control.

The reasons for opinion changes were not explicitly ascertained in this study. Other studies have found that the provision of general information [31] or information about the impact of selection bias [8] may be important in changing opinions toward greater acceptance of use of deidentified data without explicit consent. Other studies have shown that, during focus groups, people change their mind to become more accepting about such data use, rather than the reverse [11,32]. However, this study also found that, for a minority of people, their opinion changed toward being more skeptical about data use. From the perspective of public engagement about data use and linkage, this suggests that some individuals may well receive the same information but reach different conclusions to their peers, perhaps by applying different values. It also calls into question the assumption that public distrust will necessarily be addressed, such as the deficit model of public understanding of science, by simply providing greater dissemination of information [33,34].

Many of the jurors changed their minds from preferring either no public input or an opt-in model to preferring an opt-out model. Hill and colleagues found a lack of consensus in the international literature on a model of consent that was preferred by the public [8]. Taylor and Taylor found, in their small-scale study, that although some people may well *prefer* opt-in models, pragmatically, they would be willing to *accept* opt-out models [29]. Our study did not test other, more nuanced, models of consent, such as dynamic consent [35], which enable people to amend their choices as often as they wish, when they change their minds as to what they are willing to permit to happen with data about them.

The jurors wanted data to only be provided to organizations that could demonstrate that the primary goal for using the data was for public benefit (either for new treatments or to improve existing services). In addition, such organizations would have to be trustworthy because of their previous track record and existing controls and safeguards against misuse. This is similar to what has been found elsewhere, both nationally [11,36] and internationally [9,37]. Although commercially funded research has been considered unacceptable in some studies [8], some jurors became more willing to accept such uses by the end of the jury proceedings. This may reflect that *why* commercial research was being conducted (eg, for public benefit) mattered more than who was conducting it [11].

### Use of Citizens’ Juries

As has been found in other studies [38], this work shows that citizens are capable of critically evaluating expert opinion presented to them, identifying and seeking out any additional knowledge they need by asking questions from the witnesses, and then using deliberation to reach an agreed opinion. Such deliberation went on throughout the jury process, as was seen when jury members described changing opinions following discussions with their peers and the differences seen in the prejury and postjury questionnaires.

These citizens’ juries were conducted as close as possible to the ideal suggested in a recent systematic review [18]. Our links with the Jefferson Center and having a member of their staff act as lead jury facilitator (KB) ensured that the citizens’ juries demonstrated the three important characteristics of deliberative democracy: inclusivity, deliberation, and active citizenship [18]. The selection criteria ensured that people from a broad range of backgrounds were recruited, including those with opposing opinions on privacy and whose voices might not otherwise be heard [39]. The recruitment method was different from that found in the Jefferson Center manual, which advocated cold calling random telephone numbers [13]. Even in 2004, the authors cautioned that 180 calls would be needed to recruit each juror. Online recruitment and completion of a screening questionnaire was a 21st century update (approved by the Jefferson Center) that proved much more efficient, as it required little staff time to identify potential jurors.

By paying jurors appropriately for their time, it was possible to ensure that participants were representative of the population and not limited to the subgroup of people with sufficient resources to enable them to engage in a lengthy volunteer activity. Citizens’ juries use panels that are selected to be representative of the population [13]. The Jefferson Center manual highlights that criteria should be both demographic and attitudinal, with targets based on existing data. Hence, we used a national census for criteria based on demographic data [19] and a recent survey of public opinion on balancing privacy against information sharing for privacy base criteria [20]. We acknowledge that this produced a jury with more people in favor of data sharing, but this reflects the views of the overall British population. In a small sample of 18 people for each jury, it was important that we did not by chance recruit a disproportionate number of people who were very supportive of information sharing, or a disproportionate number of people who were very privacy conscious and cautious about information sharing.

The witnesses presented diverse viewpoints, and adequate time was allowed for jurors to question and challenge the witnesses. More than half the available time was given to jury deliberation, which was conducted in small groups with varying participants, to ensure that jurors interacted with all others. The evidence from the videos shows the care with which the jurors approached their role. The jurors were told from the beginning that the findings from the juries would be fed back to policy makers to ensure active citizenship. Twelve jurors (6 jurors from each jury) were invited to the postjury workshop where they successfully engaged with the invited stakeholders, including national policy makers.

The findings from citizens’ juries are qualitative in nature, and therefore, the findings are not intended to be generalizable in a statistical sense. As described by Lincoln and Guba [40], qualitative research aims for transferability by showing how the findings may be applicable to other contexts. To achieve this, the jury process and materials have been published on the Web [26], alongside this paper, to increase transparency and allow other readers to consider whether the findings are applicable in broader contexts.

Bias, both conscious and unconscious, is an important criticism of citizens’ juries [41]. Despite the efforts of the researchers and the independent oversight panel, to ensure that jurors were presented with balanced information to ensure overall fairness, some jurors reported a perception of bias. The witnesses had been chosen so that one set were intentionally impartial and one set were intentionally partial and aiming to present a particular side of the arguments to the jurors relative to the mission. There may have been a lack of clarity about this for several jurors, which could have led to these impressions. This suggests that bias in citizens’ juries can be monitored and minimized, but not totally eliminated.

It could be argued that deliberative methods should be used for making complex policy decisions. There is some evidence from the literature that people are happy to have an ethics committee make decisions about whether to approve the use of data for individual research studies [42]. In addition, there have been centuries of experience of using a 12-person jury in criminal trials. It has been suggested that citizens’ juries *symbolically* represent the community [41,43]. Nonetheless, citizens’ juries such as the ones conducted here are not usually given public accountability for their decisions and therefore, may be less acceptable to members of the general public.

From a practical perspective, however, deliberative methods such as citizens’ juries are not a reasonable choice for all policy decisions because they are so resource-intensive. They could be a good choice for situations where the topics are both important and potentially intractable or where democratic legitimacy is needed for decisions [44]. In addition, there is no expectation that the methodology should (or indeed could) be scaled up to provide a large-scale public engagement activity itself. Alternative activities that are designed to reach large numbers of citizens, however, can be informed by the knowledge gained from having previously conducted citizens’ juries.

### Conclusions

Our citizens’ jury method was successful in enabling members of the public to deliberate and make decisions about a complex policy problem. Many jurors became less skeptical about health data sharing, as they became better informed of its benefits and risks. Most jurors wanted public input in the form of information provision and the right to opt out. This was one of only a few studies to show that during a deliberative process, a small minority of people become more skeptical about data use, rather than less. This suggests that public engagement about the data use cannot assume that merely providing more public information will equal more public trust.

The deliberative method used in this study may help uncover often-overlooked opportunities for policy makers to engage meaningfully and substantively with the public about technical, and potentially divisive, public policy issues—especially those that have been recently controversial. This research demonstrates that citizens’ juries can be an effective model for engaging the public on policy issues that balance competing issues such as potential risks to individuals, the pursuit of commercial profit, the search for answers to research questions by academic institutions, and the possibility of direct public benefit for society as a whole. Further research is needed as to whether citizens’ juries would be acceptable to the public as a way to have an informed set of peers make decisions on their behalf.

## Appendix

Multimedia Appendix 1 Presentations from expert witnesses.

Multimedia Appendix 2 Main reasons given by juries for their votes for part 1 of jury mission from the jury reports.

## Abbreviations

ICO: Information Commissioner’s Office
NHS: National Health Service
