# Supplementary material for: Investigating the Extent to Which Patients Should Control Access to Patient Records for Research: A Deliberative Process Using Citizens’ Juries
Source: J Med Internet Res. 2018 Mar 28;20(3):e112. doi: 10.2196/jmir.7763 (PMC5895919; doi:10.2196/jmir.7763)
Supplement: Multimedia Appendix 1 [file jmir_v20i3e112_app1.pdf]

8. Expert witnesses

Dr Ralph Sullivan is a general practitioner with a specialist interest in patient records and systems. He is here to explain what is in a patient record, and how patient records are used in the NHS.

Dawn Monaghan works for the Information Commissioner's Office, and her responsibilities include the protection of health records. She is here to tell you a little about the law that protects access to patient records.

Dr John Ainsworth is a researcher at the University of Manchester, and the deputy director of a centre that uses information technology and health records to improve the health of the population. He is here to argue that it's important that patient records are used for research and other purposes that bring benefits to the public.

Sam Smith works for medconfidential, an organisation that campaigns for patient confidentiality, and better protection of patient records. He is here to make the case for stronger control over access to patient records, and better information and choices for patients about the use of patient records.

Prof Soren Holm is a medical ethicist at the University of Manchester. Soren will present ethical arguments for patients controlling access to patient records, and ethical arguments for wider use of patient records for the benefit of the public.

Dr Ralph Sullivan

Patient Health Records

Their Contents and Use

I am going to explain …

- What is in NHS patient records
- Who keeps them
- How are they used in personal healthcare

- What else patient records are used for
- Consequences if they were not used for these other purposes
- Reasons for using information that might identify patients

Paper to Computers by 2020

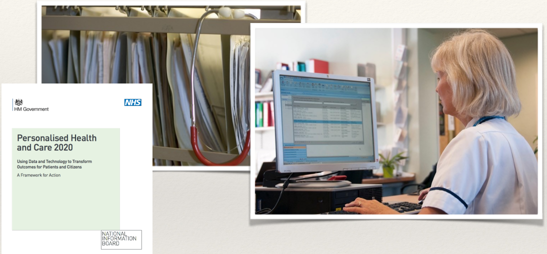

"All patient and care records will be digital… by 2020"

General Medical Council

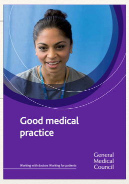

21. Clinical **records** should include:
    - relevant clinical findings
    - the decisions made and actions agreed, and who is making the decisions and agreeing the actions
    - the information given to patients
    - any drugs prescribed or other investigation or treatment
    - who is making the record and when

20. You **must** keep records that contain personal information about patients, colleagues or others **securely**, and in line with any data protection requirements.

50. You **must** treat information about patients as **confidential**. This includes after a patient has died.

Medical records - building blocks

- Identity data
- Free text - notes
- Coded machine-readable
- Images
- Letters and reports

- Important illnesses, accidents, operations
- Biological data (e.g. BP, wt)
- Investigations and screening tests
- Prescribing and treatment
- Lifestyle and social circumstances
- Appointment data

Patient record storage

Data controllers<br>NHS organisations	Data processors<br>Commercial companies

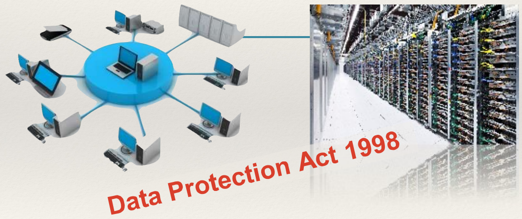

Antibiotic prescribing audit

Parent Population: Co-amoxiclav prescribing
Last Run: 12-Aug-2014 13:46 **Relative Date**: 12-Aug-2014 13:46

Patient Count	Males	Females
42	18	24

Patient Details	Medication Issues			
EMIS Number	Name, Dosage and Quantity	Date of Issue	Issuing User's Surname	Linked Problems' Code
15322	Co-amoxiclav 500mg/125mg tablets	08-Jul-2014	McKirdy	
15996	Co-amoxiclav 500mg/125mg tablets	14-Apr-2014	Parkinson	Dog bite
16151	Co-amoxiclav 250mg/125mg tablets	21-May-2014	Grant	
16385	Co-amoxiclav 500mg/125mg tablets	20-May-2014	Sullivan	Diverticulitis
17375	Co-amoxiclav 500mg/125mg tablets	04-Aug-2014	McKirdy	Diarrhoea
17768	Co-amoxiclav 500mg/125mg tablets	02-Jul-2014	McKirdy	Abdominal pain
20155	Co-amoxiclav 500mg/125mg tablets	23-May-2014	Lister	Urinary tract infection,
20566	Co-amoxiclav 500mg/125mg tablets	14-Jul-2014	Astle	Tonsillectomy
20687	Co-amoxiclav 500mg/125mg tablets	01-Apr-2014	Disberry	[X]Bite from human
21546	Co-amoxiclav 500mg/125mg tablets	27-May-2014	Morgan	Animal bites
21989	Co-amoxiclav 500mg/125mg tablets	15-Apr-2014	Astle	
22090	Co-amoxiclav 500mg/125mg tablets	28-Jul-2014	McKirdy	Chronic obstructive pu

One patient, many records

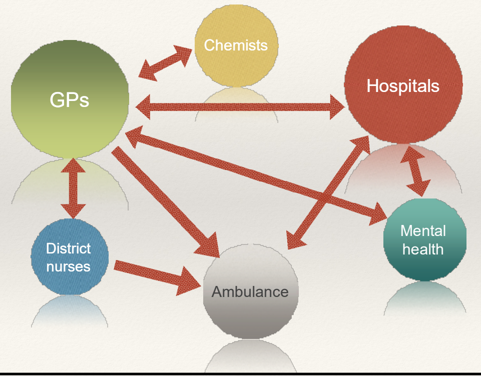

Health Record Viewers

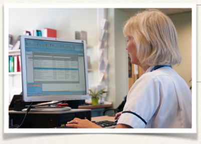

Healthcare Gateway

- GP records in A&E in Morecambe Bay
- "Out of hours" record viewer in North Lancashire and Cumbria
- Extended hours GP service in Wigan
- Patient Access nationwide

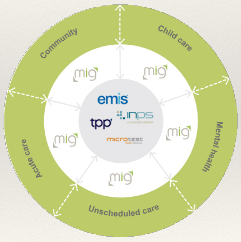

Other uses of health data

- NHS payment system
- Quality monitoring
- NHS planning and public health

- Research

- Non-healthcare uses

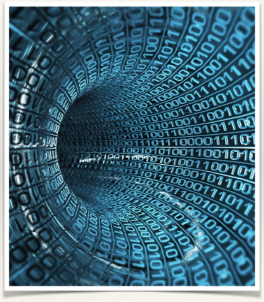

New records - Q1

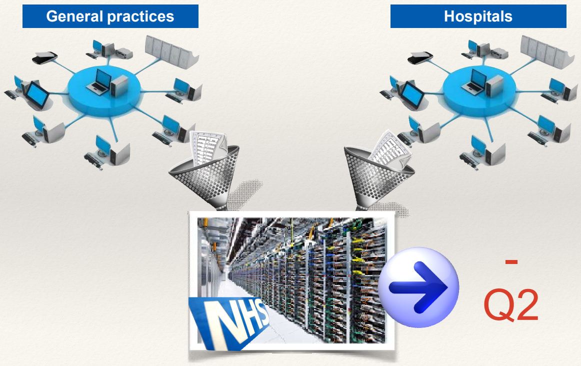

- Q2

De-identifying patient level data

Patient-level records for direct care include …

- Name and address, email address, telephone numbers
- **NHS number**, date of birth, post code, gender

Protecting identity in the new record

- Remove identifiers, blur or remove data
- Physical security and encryption
- Data sharing agreements

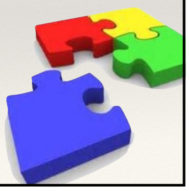

Examples of secondary uses

- Secondary Uses Service (SUS)
- Hospital Episode Statistics (HES)
- QResearch
- Office of National Statistics (ONS)

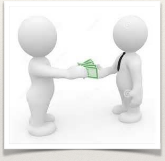

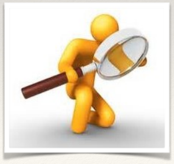

Secondary Uses Service (SUS)

- When a patient or service user is treated or cared for by hospitals, information is collected which supports their treatment.
- SUS is a data warehouse containing this identifiable patient-level information from NHS Trusts in England.
- NHS providers and commissioners can use the data for 'secondary purposes':

- Healthcare planning
- Commissioning services
- Payment by Results
- Improving public health
- Developing national policy

DIABETES UK
CARE. CONNECT. CAMPAIGN.

National Diabetes Audit

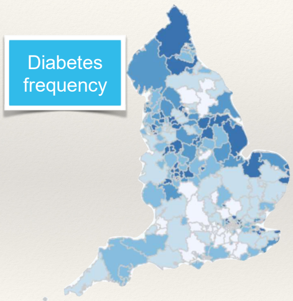

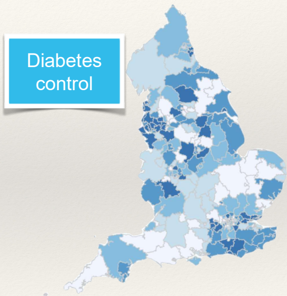

Summary

- NHS organisations keep extensive health records
- Identifiable data is shared for your direct care
- New records are made by combining records from different NHS organisations
- Many organisations ask for health data for purposes that may benefit you indirectly
- Patient level data is often needed

Thank you

Citizens Jury

Dawn Monaghan, Group Manager, Strategic Liaison ICO UK

ico.
Information Commissioner's Office

Can a public body legally create records about you?

- Yes
- Any organisation can potentially create a record which contains information about you
- There are legal requirements to abide by and rules which must be met

ico.

What must organisations do when they create a record?

Follow the Law

To do that they must;
- Maintain a duty of confidence
- Meet the principles of the Data Protection Act
- Follow the spirit and the articles of the Human Rights Act

- The key point is that organisations have to handle your information in line with the law and there are some restrictions on what they can do and how they must do it.

ico.

Confidential Information

- When you provide a professional or an organisation with information about your health you usually have an expectation it will remain private.
- You may recognise that others providing treatment to you will see the information but would expect it to otherwise be kept private.
- Organisations have a duty to ensure that happens.

ico.

What protection does the DPA offer?

- Protects information about you (that identifies you) if you are still alive!
- It doesn't apply to anonymised data – This is data which has been de identified
- What about the grey area in the middle?!
- Linked data, the risks/likelihood of re identification, the content and context - jigsaw effect
- Safeguards – protection if re identified

ico.

Protecting the information

- Organisations can only collect and use data when it is legitimate, fair and secure to do so.
- They must have systems in place to make sure the information is up to date and relevant to the purpose they are using it for.
- They must protect the information from unauthorised use and hacking.

ico.

Can an organisation create a record without asking me or telling me?

- Yes, in many circumstances they can create one without asking you.
- They don't always have to ask but almost always they do have to tell if they are using information about you (that which relates to you and if you are still alive!).
- Organisations are bound by law to tell you what they are doing with information about you, why and how they are doing it whom they are sharing it with.
- How should they/do they tell you?

ico.

How do organisations protect the information?

Some of the things the practical things they have to do are;

- make sure the information they have is correct
- put technology in place which allows the data to be protected properly
- train the staff to be able to handle personal data properly
- Be able to provide the information they have which is about you to you on request

ico.

Who can have access to the record?

- Several organisations could be allowed access to the record commercial NHS etc.
- In deciding to share the information the organisations must follow a Data Sharing Code which sets out how the information should be handled.
- It is the responsibility for the originating organisation to make sure that the receiving organisation/s can legitimately, fairly and securely gain access and use the information.
- If anonymised it is done so to a specific standard.
- Once the receiving organisation has been given access they must also ensure the data is handled in an appropriate way i.e not re identified or protected.

ico.

Who owns the information in the record?

Patient Records

- Not all of the information contained within the record may be information about you.
- Some information may relate to another person.
- Some information may be medical opinion.

ico.

Who owns the information in the record?

- The law doesn’t state that anyone is the owner of the personal information held by an organisation.
- You may ‘own’ the information before you provide it to an organisation.
- Once they have it you have some protection in regard to how it is used, but you don’t ‘own’ the actual information or the patient record.
- The organisation doesn’t ‘own’ the information either, but they do have responsibility for it.

ico.

What rights do you have?

Some of your rights are;

- To have a copy of the information which relates to you (such as a copy of your patient record) provided to you on request;
- in certain circumstances have inaccurate personal data rectified, blocked, erased or destroyed (not always opinions); and
- complain to the Information Commissioners Office if you feel the data has been handled incorrectly.

ico.

Who holds the organisations to account?

- The Information Commissioners Office
- Department of Health/NHSE/HSCIC
- The role of the National Data Guardian
- Professional standards bodies and regulators – GMC, CQC etc

ico.

In summary

- All organisations can potentially create, hold, use and share information about you
- Neither you nor the organisation 'owns' the information in the record
- The Data Protection Act provides protection for any information which identifies you (if you are alive!)
- An organisation doesn't always have to ask your permission but they almost always have to tell you what they are doing
- You have certain rights
- Several organisations have responsibilities to ensure the organisations using information about you are acting in a legitimate, fair and appropriate way

ico.

Dr John Ainsworth
Health eResearch Centre & Farr Institute

1. **Five questions we ask about care**
2. What is evidence?
3. What is good evidence?
4. How to respond to the charge

Five questions about your healthcare

- Is it safe?
- Does it work?
- Is it value for money?
- Is it acceptable?
- Is it fair?

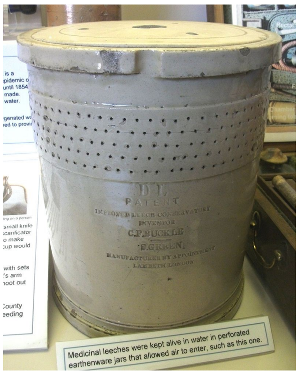

"Leech Jar Bedford Museum" by Simon Speed - Own work. Licensed under Public Domain via Commons

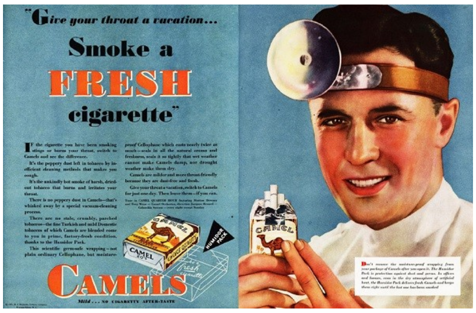

1. Five questions we ask about care
2. **What is evidence?**
3. What is good evidence?
4. How to respond to the charge

History of Evidence

- Past - Folk medicine
  - Treatment
- Today - Evidence based medicine
  - Disease + treatment
- Future - Personalised medicine
  - Person + disease + treatment

What is evidence in healthcare?

When we use data to answer our questions

- Is it safe?
- Does it work?
- Is it value for money?
- Is it acceptable?
- Is it fair?

1. Five questions we ask about care
2. What is evidence?
3. **What is good evidence?**
4. How to respond to the charge

Marbles in a bag

- I have a bag with 60 million marbles in it
- A marble may be red, green or blue
- I want to know how many in the bag are red, green or blue
- How do I do it?

Marbles in a bag – Solution 1

- Pull out a fraction of the marbles and scale-up
  - I pull out six marbles
  - four are red, one is blue, one is green
  - Scaling up by 10m gives … 40m red, 10m blue, 10m green ... making 60m in total
- Next time I repeat the experiment I pull out 6 blue marbles and get a very different answer!

Marbles in a bag

- So how many do I need to pull out to get an accurate answer?
  - 6
  - 60
  - 600
  - 6000
  - 60000
  - 600000
  - 6000000
- The more marbles I pull out the more reliable the answer, at the cost of time and money

Marbles in a bag – Solution 2

- Pull out every marble and record the colour
  - Exact answer
  - Very time consuming and expensive
  - Impractical to repeat
    - What if the marbles are either small, medium or large
    - What is the marbles are made of wood, glass or metal
    - etc …
- What would happen if all the red marbles were removed from the bag before we started?
  - Our answer would be very wrong

About health records

- Across the whole of our population we each have a health care record
  - Compare with knowing about each marble in the bag
- Our health records already record detailed information about us
  - Compare with knowing the colour, size, material of the marbles in the bag
- Removing access to records is like removing marbles from the bag before we start counting

Where does evidence come from?

Solution 1

By undertaking highly controlled clinical trials, case-control studies or prospective studies.

PROs – informed consent, high quality data, best evidence

CONs – does not generalise, slow, expensive, narrow scope

Solution 2

By analysing healthcare data collected throughout standard clinical practice

PROs – large numbers, timely, broad, already exists

CONs – not consented, low data quality, potential for confounding

Rofecoxib

From Wikipedia, the free encyclopedia

Rofecoxib /ˌrɒfɪˈkɒksɪb/ is a nonsteroidal anti-inflammatory drug (NSAID) that has now been withdrawn over safety concerns. It was marketed by Merck & Co. to treat osteoarthritis, acute pain conditions, and dysmenorrhea. Rofecoxib was approved by the Food and Drug Administration (FDA) on May 20, 1999, and was marketed under the brand names **Vioxx**, **Ceoxx**, and **Ceeoxx**.

Rofecoxib gained widespread acceptance among physicians treating patients with arthritis and other conditions causing chronic or acute pain. Worldwide, over 80 million people were prescribed rofecoxib at some time.[1]

On September 30, 2004, Merck withdrew rofecoxib from the market because of concerns about increased risk of heart attack and stroke associated with long-term, high-dosage use. Merck withdrew the drug after disclosures that it withheld information about rofecoxib's risks from doctors and patients for over five years, resulting in between 88,000 and 140,000 cases of serious heart disease.[2] Rofecoxib was one of the most widely used drugs ever to be withdrawn from the market. In the year before withdrawal, Merck had sales revenue of US$2.5 billion from Vioxx.[3] Merck reserved $970 million to pay for its Vioxx-related legal expenses through 2007, and have set aside $4.85bn for legal claims from US citizens.

Rofecoxib was available on prescription in both tablet-form and as an oral suspension. It was available by injection for hospital use.

Rofecoxib

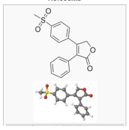

Rofecoxib, https://en.wikipedia.org/w/index.php?title=Rofecoxib&oldid=691615884

1. Five questions we ask about care
2. What is evidence?
3. What is good evidence?
4. **How to respond to the charge**

The use of health records

- Must not exploit the population
- Must be open and transparent
- Must be for the public good

1. Should the NHS body be allowed to create these records about you and other patients?

- Yes – the evidence to improve healthcare already exists
- Yes – it is in the public interest
- Yes – it should be done for the entire population to ensure the maximum benefit is gained

2. Given your answer to question 1, who should be allowed to access and extract data from the records created?

- Is it in the public interest?
- Does it benefit the population?
- Does it improve health care by answering:
  - Is it safe?
  - Does it work?
  - Is it value for money?
  - Is it acceptable?
  - Is it fair?

Citizen Jury:
To what extent should patients control access to patient records?

Sam Smith
coordinator@medconfidential.org

What do we do?

Every data flow should be:

- **Consensual**
- **Safe**
- **Transparent**

medConfidential KEEP MY SECRETS

This is how we look at everything. So if you want to have a nap, you’ve got the key points.

{extend on all 3. not covering safe here - don’t leave papers on trains etc. Safely in theory? Safely in practice?}

Who am I?

Background in data & social science academic research

Data and technology

I was a data person working in a research group for a decade. First in academia, then at Privacy International, and for the last 2 and a bit years, at medconfidential.

We all like research. This is a research project after all. You could have said no, and you can change your mind (but Malcolm would appreciate if it you didn't) at any time.

The thing about consent for data, is when it's gone, it's gone. it's hard to get back.

What is the data?

- linked - by the NHS number
- lifetime - from birth to death
- detailed - everything in between

just to recover things you've probably been told already.

{maternity/birthday problem; 2 birthdays; 90% likely identification; 3 birthdays, effectively unique}. Particular conditions for various reasons.

Why should data be confidential?

- You
- Your family
- Your community
- Specific risk groups

Who decides what others know?
How much of the record is shown?
Gossip. Who trusts their GP not to gossip? Who trusts their GP receptionist not to gossip?

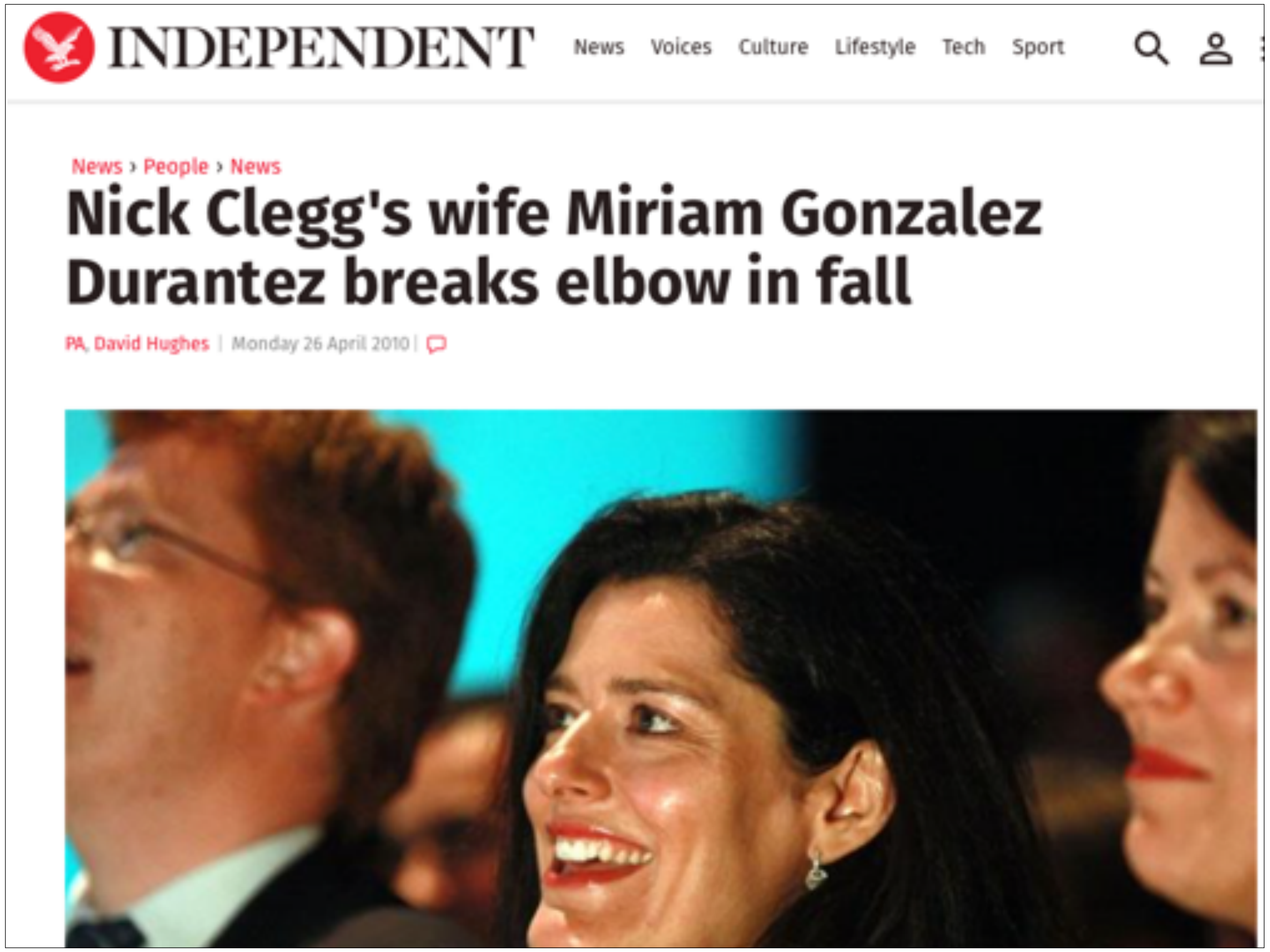
INDEPENDENT News Voices Culture Lifestyle Tech Sport

News › People › News

Nick Clegg's wife Miriam Gonzalez Durantez breaks elbow in fall

PA, David Hughes | Monday 26 April 2010 |

Election 2010. Nothing else happened that day, so every newspaper wrote about how Mrs Clegg fell over, and there were lots of press articles about it and the hospital, as nothing else happened that day.
How many 42 year old women broke their elbow in that way on that date in that hospital? Linked, lifetime, medical history.

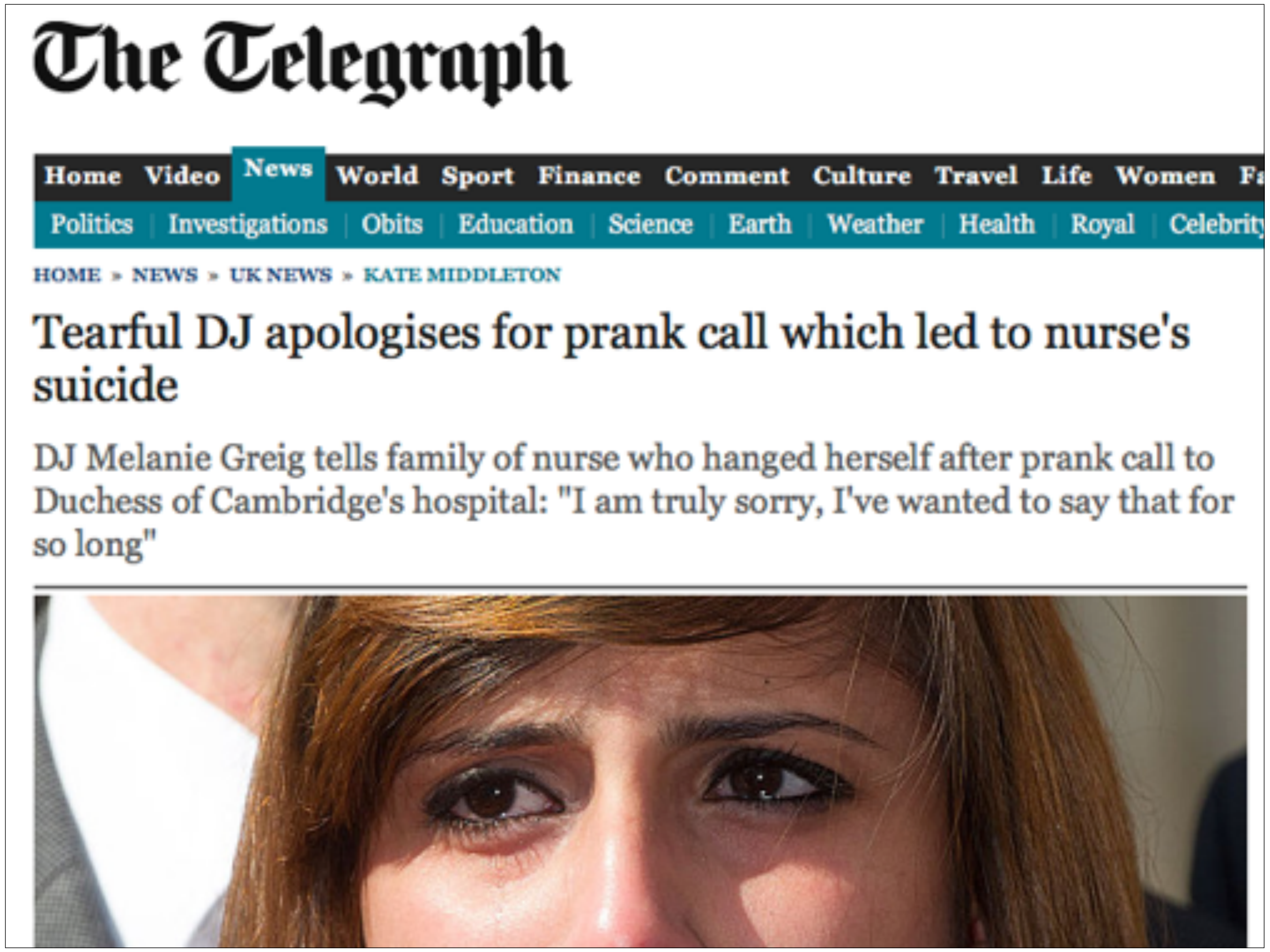
The Telegraph

Home | Video | News | World | Sport | Finance | Comment | Culture | Travel | Life | Women | Fa

Politics | Investigations | Obits | Education | Science | Earth | Weather | Health | Royal | Celebrit

HOME » NEWS » UK NEWS » KATE MIDDLETON

Tearful DJ apologises for prank call which led to nurse's suicide

DJ Melanie Greig tells family of nurse who hanged herself after prank call to Duchess of Cambridge's hospital: "I am truly sorry, I've wanted to say that for so long"

{harms - this is a fully linked dataset. It's an information problem.}
It's everything in your medical history. Whose choice is that?
Are there things your wife wouldn't want you to know?
Are there things you don't want your ex-husband to know?
Critically, what about the gossipy neighbour two doors down who works for the organisations you're dealing with.

Consent choices

- **Medical treatment is only given with your informed consent. You can always say no.**

- **implied consent for data?**

medConfidential
KEEP MY SECRETS

Opt out: “Do the right thing”

- “implied consent”
- opt out is the current basis
- opt in? to what?

medConfidential KEEP MY SECRETS

There are often two worlds “Opt in” and “Opt out”

An “opt out” model is where there’s a decision on what the “right thing” is, everyone gets told, which the ICO talked about yesterday, and those who disagree can “opt out”.

Research example: pharma journalists, domestic abuse survivors

Opt in, is opting in to a specific list of specified things. This is how surgery is done - you give informed consent to a particular thing. However, “opt in” for data for the very long term is hard. How do you write that so it’s understandable, specific, and sustainable?

Research and NHS management

- **Will take as much data as they can have.**
- **Will always want more.**

The data is never enough. If it was, it wouldn't be research or micromanagement.

Research ethics: only do research on people willing to be researched.

If you get the question right, 98% of people are happy to help. Just like you are.

Commercial use

- **Commercial use is use of data by commercial entities for their benefit**

medConfidential
KEEP MY SECRETS

What about commercial use?

You have 2 commercial uses on your list

Commercial Re-use

- **Commercial reuse is the onward sale of data by commercial entities to other commercial entities**

medConfidential
KEEP MY SECRETS

the two entities on your list might also be “re-use”. Given the middlemen and intermediaries in both industries, the data may not go to a pharma or insurance company, they may go to a company working with them. “Data brokers” if you will.

Decommissioning

- Consent?
- Individual level records?

On NHS management, should individual level data be used for what the NHS calls “decommissioning”? (that’s closing hospitals). How would that work in an opt in or your opt out world?

There are some things individual level data shouldn’t be used for — those purposes are met with aggregated statistics, counts, averages, not individual data. Your first point is about funding decisions — has a case been made that they require **individual** level data? Or do they just need to know summaries, averages, “people being treated for this condition in this hospital do 10% better than that one?”. Creating the statistics requires individual level data, but the statistics are published, in the same way statistics on the economy are published, but your bank statement isn’t.

How much do you want to know?

Currently, how your personal medical record is used is secret.
All of the scenarios you've been asked to consider might happen. None of them might. You don't know.
Should you know how individual level data about you has been used?

Experience

it's shorthand to say that opportunities get used by "fraudsters and charlatans" first.
There's an NHS Pharmacy called Pharmacy2U, they're an internet chemist, which do prescriptions over the web, and will sell you little blue pills, apparently discreetly.
Then the ICO found out that they sold the names and addresses of their customers without telling them.
Whether they should have done that is not our concern.
It's who was at the front of the queue that's most of interest, and what they wanted. They weren't marketing things to 20 somethings buying little blue pills.
They were marketing to people who were elderly, who were less congnitively aware.
One was selling herbal replacements for the prescriptions they'd just bought. They didn't work, but they were expensive.
Another was running a lottery scam, going after life savings.
The people targeted when data gets sold are the elderly, the less cognitively aware.
And if you think that doesn't affect you, if your mother in law loses her life savings due to a scam, where's she going to live? ;)

Predicting the future

the framework has to work into the future.
The job of the NHS is to save lives.
Whatever happens, the decisions you take have to work for everyone. Those keen for their data to be shared, and those who have good reasons for it not to be.

Questions

Every data flow should be:

- **Consensual**
- **Safe**
- **Transparent**

Questions?

Public Benefit and Individual Interests in the Secondary use of Health Data

Søren Holm
University of Manchester

Overview

1. The Problem: Public benefit versus Individual interests
2. Public benefit – What and why
3. Individual interests – What and why
4. Can the interests on both sides be reconciled
5. Conclusion

The problem

Health information is generated in individual clinical encounters between health care professionals and patients. This usually happens under conditions of confidentiality.

But,

1. it is necessary for the health information to be used outside of the clinical setting, e.g. for public health, administrative and planning purposes.
2. important health research can only take place if researchers have 'easy' access to large, complete datasets of health information.
3. There are other potential uses that may also generate public benefit even though they are carried out by private firms.

Public benefits

Research:
Well conducted health research produces public benefits.

Some of the public benefits are economical, but the main benefits are better understanding of health and disease, the causes of disease, the effectiveness of treatments etc.

'Industry':
There is a potential public benefit in effective insurance underwriting, evidence based planning of pharmaceutical research portfolios etc.

Many of the benefits come from linking information from different sources. This linkage can be performed in ways so that researchers only have access to anonymous datasets.

Public and individual interests

A public interest is an interest in securing a public good, which is not necessarily the same as something that the public is interested in

> There is a public interest in road safety, but not a public interest in Peter Andrés love life (despite some segments of the public being interested in the latter)

There is an overlap between public and individual interests.

- As citizens we all have an interest in health research taking place, i.e. we all have an interest in the progress of medicine and in the welfare of our fellow citizens
- It could also be argued that if we willingly use the NHS, then we have an obligation to contribute to the NHS and allow our health data to be used for health research

Individual interests in protecting and controlling health data

In relation to identifiable information:

- Confidentiality*
- Privacy
- Individual harm caused by disclosure

In relation to anonymous information:

- Control over use
- Group harm

**Important to note that there is also a strong public interest in confidentiality being maintained in health care*

Specific informed consent is not the solution

We could require that individuals should be asked for their informed consent every time their data are used for health research (or for non-research purposes by industry)

This would, in theory enable them to protect their own interests by consent or refusal

But, this is in reality not a good solution. It is bad for research and does not really protect individuals:

- It would be very costly
- It would make research very difficult
- We need to question whether the consent would be valid:
  - Would people really think about the issues
  - Would we get 'routinisation of consent', i.e. will a person really think carefully about their decision when they are asked for the 10th time in a year

Can the interests be reconciled 1?

Identifying interests is not enough, we also have to decide their 'weight'

Individual interests are stronger / more weighty in relation to the use of identifiable information than in relation to the use of anonymous information

Individual interests may be stronger in relation to specific types of information (e.g. about mental health or sexually transmitted diseases), or specific research questions (e.g. related to race or religion)

Public interests in research are stronger the more important the research question is ≈ the more important the health problem that is being investigated is

Can the interests be reconciled 2?

Is individual control the only way forward, or are there other tools in the toolbox?

- Data security requirements
  - Strict access control with logging of users
- De-identification
  - Datasets with minimal possibility for re-identification
  - Anonymous linkage
- 'Licensing' of researchers
  - Sanctions if rules are breached
- Body to approve research proposals
  - Different levels of requirements depending on sensitivity of data and sensitivity of research question
- Making information easily available about ongoing research

We could also consider more sophisticated ways of getting individual consent using modern ICT technologies

Further on 'industry' use for activities that are not health research 1

Perhaps additional worries about:

- Is the activity really in the public interest?
- Will agreements about restricted use of data be kept?
- Will the NHS be tempted (or compelled) to sell data for purely commercial purposes, e.g. to enable better targeted advertising of health related products?

Further on 'Industry' 2

These concerns are not theoretical and fanciful, but the best way to guard against systematic misuse of data is not individual, specific consent, but strong regulatory mechanisms

Conclusion

Most health research using anonymous health data does not significantly go against the interests of individuals

If appropriate strong safeguards are introduced , individual specific consent is only needed in circumstances where research is especially sensitive because of the type of data used, the research question, or because it uses identifiable data
