# Supplementary material for: Investigating the Extent to Which Patients Should Control Access to Patient Records for Research: A Deliberative Process Using Citizens’ Juries
Source: J Med Internet Res. 2018 Mar 28;20(3):e112. doi: 10.2196/jmir.7763 (PMC5895919; doi:10.2196/jmir.7763)
Supplement: Multimedia Appendix 2 [file jmir_v20i3e112_app2.pdf]

Appendix 2. Main reasons given by juries for their votes for part 1 of jury mission from the jury reports.

	**Jury 1**	**Jury 2**
Vote 1A. Should the NHS body be allowed to create these records about you and other patients?		
Yes	• More detailed and complete data can produce more accurate evidence which can lead to more effective, more cost effective health care through NHS. Including more records and information produces more reliable data. Utilizing more data can improve healthcare for a larger share of the population. Improved health care delivery would serve NHS in improving the public good and public health<br>• Personalized medicine and treatments can only be discovered and utilized effectively through use of more complete data and records<br>• The data already exists in our current personal health records and consolidating would make it easier to access and use for research<br>• Creating new records and sharing records can help identify ineffective drugs and treatments sooner so that they can be removed from use	• The more data available for analysis, the stronger and more reliable the evidence, results, and outcomes. Projections and estimates will be more accurate if organisations are able to utilize a single, unified dataset for their analyses<br>• More records included in datasets will allow better monitoring of treatments and prescription drugs, increasing patient safety<br>• Creating and sharing a record created from GP and other records will be more cost-effective for organisations and potentially reduce duplication of records, increasing the quality of data available for analysis<br>• People and organisations are already using data and records for research, analysis and other purposes – creating a record from GP and hospital records may make this more efficient for researchers

	**Jury 1**	**Jury 2**
No	• Data may be used by private companies for commercial gain rather than for the benefit of patients and the public<br>• There are significant concerns about data transparency, including who has access to and control of the data and records, especially people, companies or organizations who use the data for purposes that are unclear or other than what we authorize<br>• Some people have an increased risk of having information accessed by someone they do not authorize (eg., a partner, spouse, former contact) who has access to data or records through their position<br>• Inadequate sanctions or punishments for breach of the Data Protection Act (DPA) may not deter organizations from misusing records	• Without a clear understanding of who will be regulating the data and making decisions about access it is difficult to support the creation of new records<br>• Despite safeguards, data and records may not be secure and may be accessed by individuals and/or organisations who don't have proper permission or legal authority<br>• Commercial reuse of records or data does not necessarily have a public benefit and may be used by organisations to increase profits or for marketing purposes<br>• It is unclear whether or not these records will be kept continuously up to date
Vote 1B. If such records were created should they be published or allow patient input		

	Jury 1	Jury 2
Publish only	• This will ensure more accurate, complete data when all records are included<br>• This will save time and money through a much more streamlined, efficient process<br>• Saving and improving lives takes precedence over concerns about data sharing. There is a qualitative difference between people who may be upset or inconvenienced by data breaches and those who may die or not receive better treatment because the data is not available<br>• This removes the need or requirement for individuals to make a decision without being properly informed	• Having a more complete data set will be a greater benefit to the population and would serve the greater good<br>• Based on the time and expense for providing an option for patients to participate, this approach will save time and money and improve results<br>• The process of getting truly informed consent from a large percentage of the population is too costly and too complicated - many are likely to make an opt-in or opt-out decision without clarity<br>• Data collection and research are likely to take place regardless and organizations shouldn't have to justify why they are doing their research to individuals if they have been approved by another body
Patient option	• People should be able to have control over their own data and records<br>• This will allow greater transparency in how records are used and shared<br>• An option will allow individuals to maintain their confidence in doctors and other healthcare settings where trust is critical<br>• An option allows individuals to retain their autonomy and supports equality in how the NHS operates	• As part of a democratic process it is important for each person to have autonomy and freedom of choice when it comes to their own data or records<br>• Individuals should have a choice as to whether or not their record is included because it pertains to their own, personal information<br>• Providing the option to have their info included will inform and educate the general public about how their data is being used<br>• Not providing individuals a choice regarding how, by whom, and for what purpose can lead to intense dissatisfaction from the public
Vote 1C. Should individuals have the option to opt-in or opt-out?		

	**Jury 1**	**Jury 2**
Opt-in	• Those who choose to opt-in are clearly interested in being involved and there is no ambiguity in that decision<br>• This would allow individuals freedom to clearly choose if they want to be involved<br>• This protects vulnerable people who may not be able to understand the risks or the potential consequences of being involved<br>• If you have opt-out and it is not advertised clearly or properly, people may unwittingly join or be involved	• This option would require the body or organisation to conduct an information campaign to educate the public<br>• An opt-in option will ensure that individuals whose data are used in analysis make the intentional decision to be included<br>• This option may force people to engage with the information and learn more about the issue in order to make an informed decision<br>• People would have more control of their records and data
Opt-out	• It is easier to include individuals in a data set if they are included as a default option<br>• More people would be included in the data and this would lead to more accurate results and more representative samples of the population – this could lead to more effective research and better treatments<br>• This could be more time effective and cost effective as it is an easier, more convenient option for individuals<br>• This option is more likely to overcome apathy and doesn't make people choose about something they may not know about	• More people would be automatically included in the database meaning more data for analysis<br>• The process of adequately providing individuals the chance to opt-in would be hugely expensive and time consuming. It would take an enormous effort and may still not properly provide the opportunity to every individual to make an informed decision<br>• This option would be more cost effective and efficient – this would be a quicker, more streamlined process<br>• Opting out would provide individuals to make a decision about whether or not their records are included in the data
